# Autologous hematopoietic stem cell transplantation for relapsed follicular lymphoma: safety profile and clinical outcome in a single-center experience

**DOI:** 10.1007/s12032-014-0310-3

**Published:** 2014-11-06

**Authors:** Grzegorz Helbig, Malgorzata Krawczyk-Kulis, Anna Kopinska, Robert Liwoch, Slawomira Kyrcz-Krzemien

**Affiliations:** Department of Hematology and Bone Marrow Transplantation, Silesian Medical University, Dabrowski Street 25, 40-032 Katowice, Poland

**Keywords:** Follicular lymphoma, Autologous hematopoietic stem cell transplantation, Relapse, Outcome, Rituximab

## Abstract

Autologous hematopoietic stem cell transplantation (AHSCT) is a treatment option for relapsed and recurrent follicular lymphoma (R/R FL); however, its value in the rituximab era remains to be elucidated. To evaluate the safety and clinical outcome of AHSCT for relapsed FL, we present a retrospective series of AHSCT for 30 FL patients (17 male and 13 female) at median age of 49 years. Patients were transplanted in second or subsequent complete or partial response after at least one therapeutic line including chemotherapy and rituximab. Overall, seven patients achieved second or higher complete response (CR) at AHSCT, whereas 23 were transplanted in partial response. Median overall survival (OS) was not reached, whereas progression-free survival (PFS) was 4.8 years. The estimated 10-year OS and PFS were found to be 60 and 33 %, respectively. There was no significant difference in OS and PFS in terms of FLIPI score and disease status at transplant. Median follow-ups from diagnosis and from AHSCT were 4.9 years (range 1.5–18.4 years) and 1.7 years (range 0.03–16.5 years), respectively. Fifteen patients relapsed, and 11 out of them (73 %) died of disease recurrence and chemoresistance. At the last contact, 19 patients are alive: 12 are in CR, whereas seven patients receive salvage regimens due to active lymphoma. AHSCT for relapsed FL patients who were pretreated with rituximab remains a safe procedure with low transplant-related mortality and long-term progression-free survival in about one-third of transplanted patients.

## Introduction

Follicular lymphoma (FL) is a B-cell malignancy with an indolent clinical course and characterized by lymphadenopathy, splenomegaly and bone marrow involvement. Most patients are over 60 years at diagnosis and were found to have *t*(14;18), resulting in the overexpression of bcl-2 protein which is involved in apoptosis [1]. Most asymptomatic FL patients at early disease stage do not require treatment [2], whereas those with symptomatic and advanced-stage disease receive chemotherapy plus rituximab [3]. The value of autologous hematopoietic stem cell transplantation (AHSCT) as a first remission consolidation is to be established as the vast majority of studies were performed in the pre-rituximab era. In sum, the benefits were demonstrated in terms of progression-free survival (PFS), but not in overall survival (OS) [4]. A large number of studies have shown some benefits of AHSCT in R/R FL, but they have been conducted prior to the widespread use of rituximab [5, 6]. Nevertheless, AHSCT for R/R FL remains controversial and to date the evidence-based data are lacking. The current indications for AHSCT in R/R FL setting have been recently proposed by expert panel of EBMT-Lymphoma Working Party. The inclusion criteria for AHSCT in RR F/R are as follows: patients in first and subsequent chemo-sensitive relapse, especially those with a short duration of response to immuno-chemotherapy and high FLIPI score [7].

Herein, we present the results of our 30 patients with relapsed/recurrent FL who were performed AHSCT in our center.

## Patients and methods

### Patient selection and characteristics

Thirty patients (17 male and 13 female) at median age of 49 years (range 21–69) underwent AHSCT between 1996 and 2011. The management of patients after diagnosis followed common standards. A histological diagnosis was established by a local pathologist using immunochemistry. The disease stage was evaluated according to the Ann Arbor staging system, and International Prognostic Index for Follicular Lymphoma (FLIPI) score was calculated as published elsewhere [8]. The diagnostic workup included physical examination, complete blood count with differential, biochemistry studies, chest X-ray, abdominal ultrasonography, computed tomography of the neck, chest, abdomen and pelvis. Bone marrow biopsy was taken at diagnosis and in patients with primary marrow involvement during response assessment. Patients were eligible for AHSCT if they met the following criteria: (1) PR or second or higher complete (CR) remission after conventional immuno-chemotherapy, (2) ECOG status 0–2, (3) age <70 years and (4) adequate hepatic, renal and cardiac function. All patients signed informed consent. The clinical characteristics of patients are presented in Table 1.

**Table 1:** Patient characteristics

Parameter	FL (*n* = 30)
Male/female; no	17/13
Median age; years, range at diagnosis	49 (21–69)
Bone marrow involvement at diagnosis; no (%)	15 (50)
Stage; no (%)	
II	3 (10)
III	11 (37)
IV	16 (53)
FLIPI; no (%)	
Low	5 (17)
Intermediate	11 (37)
High	14 (46)
B symptoms; no (%)	15 (50)
Treatment lines pre-AHSCT	
1	1 (3)
2	21 (70)
3	8 (27)
Median number of treatment cycles; range	12 (6–22)
Rituximab containing regimen pre-AHSCT	30(100)
Radiotherapy prior AHSCT; no (%)	6 (20)
Median time to AHSCT; years, range	1.6 (0.7–6.5)
Disease status at AHSCT; no (%)	
CR ≥ 2	7 (23)
PR	23 (67)
Type of conditioning; no (%)	
CBV	21 (70)
BEAM	4 (13)
Z-BEAM	5 (17)
Median days of post-AHSCT hospitalization; range	25 (18–35)
Median number of post-AHSCT blood transfusions; range	2 (0–7)
Median number of post-AHSCT platelet transfusions; range	3 (0–6)

*AHSCT* autologous hematopoietic stem cell transplantation; *BEAM* BCNU, cytarabine, etoposide, melphalan; *CBV* cyclophosphamide, BCNU, etoposide; *CR* complete response; *FL* follicular lymphoma; *PR* partial response; *Z* zevalin

### Treatment

Induction chemotherapy consisted of R-CHOP (rituximab, cyclophosphamide, vincristine, adriamycin, prednisone; *n* = 12), R-CVP (*n* = 6) and CHOP/CVP (*n* = 12). Second- and third-line therapeutic options included R-ESHAP (rituximab, cisplatin, methylprednisolone, etoposide, cytarabine), R-DHAP (rituximab, cisplatin, cytarabine, dexamethasone) and R-FC (rituximab, fludarabine, cyclophosphamide) regimens. All patients received at least one therapeutic line with rituximab in pretransplant treatment. Overall, seven patients achieved second or higher CR at AHSCT, whereas 23 were transplanted in PR. Mobilized peripheral blood was the source of stem cells for AHSCT in all patients. IVE (ifosfamide, etoposide, epirubicin) was the most common regimen used for mobilization. G-CSF (granulocyte colony stimulating factor) at 10 ug/kg/day was started from day +5 until the last day of apheresis. The number of 2 × 10^6^ CD34-positive cells/kg was considered sufficient for AHSCT, but the number of transplanted CD34-positive cells was below this threshold in three patients. The apheresis product was processed, frozen to −150°C, stored and re-infused after completion of conditioning. The preparative regimens included CBV (cyclophosphamide, BCNU, etoposide) in 21 patients, BEAM (BCNU, cytarabine, etoposide, melphalan) in four, and ^90^Y-radiolabelled ibritumomab tiuxetan (zevalin) with BEAM (Z-BEAM) in five.

### Response criteria

The response to therapy was evaluated at 1, 3 and 6 months after AHSCT and 6 months thereafter using CT. CR was defined as a disappearance of all disease-related symptoms and measurable lesions for at least 4 weeks; PR was defined as a >50 % decrease in the sum of the products of the two largest diameters of all measurable lesions for at least 4 weeks. A progressive disease was defined by any increase >25 % in the sum of the diameter of any measurable lesions or the appearance of a new lesion.

### Statistical methods

The probability OS and PFS were calculated according to Kaplan–Meier method. All calculations were made from the date of transplantation. Comparisons between the variables were carried out by log-rank test. Statistical significance was defined at a *p* value < 0.05. Transplant-related mortality (TRM) was defined as death within 100 days of high-dose therapy not related to the disease, relapse and progression.

## Results

### Cell dose and engraftment

The median number of transplanted nucleated cells was 3.3 × 10^8^/kg (range 0.02–14.47), and the median number of CD34-positive cells was 4.0 × 10^6^/kg (range 1.1–26.9). All patients engrafted. The median time to neutrophil recovery was 12 days (range 10–22), and platelet count >50 × 10^9^/L was noted after median of 14 days (range 10–21).

### Adverse events and supportive care

Thirteen patients demonstrated infectious complications at the posttransplant period. Grade 3 or 4 nonhematological adverse events were not observed. Five patients developed fever with negative bacterial, and fungal cultures and mucositis of grade 1 or 2 were observed in four cases. The other complications included proctitis (*n* = 2), gastritis (*n* = 10), pneumonia (*n* = 1) and laryngitis (*n* = 1). One patient died within the first 100 days after AHSCT due to severe pulmonary infection. Fourteen patients required G-CSF to accelerate posttransplant regeneration. Median time of posttransplant hospitalization was 25 days (range 18–35).

### Outcome and prognostic factors

**Fig. 1:**
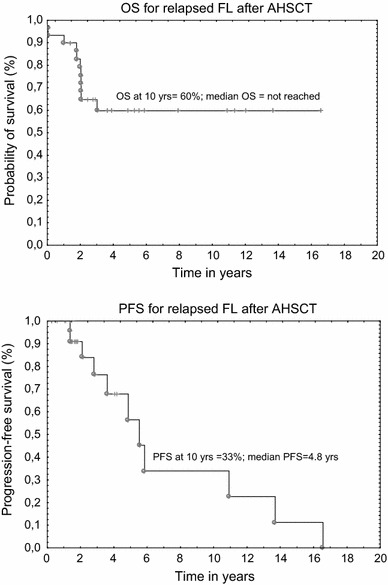
Overall and progression-free survival curves for relapsed FL after autologous hematopoietic stem cell transplantation

The TRM was 3 % at 100 day. Median OS was not reached, whereas PFS was 4.8 years. The estimated 10-year OS and PFS were found to be 60 and 33 %, respectively, see Fig. 1. There was no significant difference in OS and PFS in terms of FLIPI score and disease status at transplant. Median follow-ups from diagnosis and from AHSCT were 4.9 years (range 1.5–18.4) and 1.7 years (range 0.03–16.5), respectively. Fifteen patients relapsed, and 11 out of 15 (73 %) died of disease recurrence and resistance to chemotherapy. At the last contact, 19 patients are alive: 12 are in CR, whereas 7 patients receive salvage regimens due to active lymphoma.

## Discussion

Autologous hematopoietic stem cell transplantation can yield long-term disease-free survival when performed for FL after relapse, and this seems to be true for both patients treated in the pre-rituximab era and at the time of its wide availability [9]. However, most studies on the results of AHSCT for FL have been reported for patients who did not obtain rituximab in their induction therapy. Of note is that the vast majority of studied patients received total body radiotherapy containing regimen as a high-dose therapy. The OS and PFS at 10 years were 50 and 28 %, respectively, with ~20 % of patients being in CR 18 years after AHSCT [10]. One of the largest nonrandomized studies reported on the results of AHSCT for 248 recurrent FL patients. The preparative regimen consisted of chemotherapy in 60 % of patients, and the remaining 40 % received radiotherapy. The 5-year OS and PFS were 63 and 44 %, respectively [11]. It should be emphasized that despite the small number of included patients to our study, the OS and PFS rates were comparable with those obtained by other groups [10–12].

The significant advantage of AHSCT over standard chemotherapy for R/R FL has been unquestionably determined in the only randomized study to date. The 5-year PFS was 10 % in chemotherapy arm versus 55 % in the transplant arm; there was also a significant benefit in terms of OS in the latter one [6].

The addition of rituximab to conventional chemotherapy in FL has improved outcome; however, the plateau on PFS curves was not demonstrated [13]. Conversely, AHSCT for relapsed FL may lead to eradication of a malignant clone in a certain proportion of patients. Namely, the plateau on the PFS curve was 50 % at 7.5 years [14]. In contrast, no plateau was demonstrated by other reports [15] including ours.

It was also found that the use of AHSCT in first relapse of FL regardless of prior exposure to rituximab has ameliorated the 3-year OS [16]. A large prospective and randomized study has been recently conducted to investigate the efficacy of rituximab used both as in vivo purging before AHSCT and as posttransplant maintenance. Median follow-up of 280 enrolled FL patients was 8.3 years. There was no difference in 10-year PFS and OS between rituximab and no rituximab arms in terms of pre-AHSCT purging. The addition of rituximab as a maintenance treatment after AHSCT has significantly prolonged PFS, but no OS if compared with observation arm [17]. It remains unclear whether there is a benefit of AHSCT for R/R FL patients who received prior chemotherapy with rituximab as a first-line therapy. The incorporation of rituximab into the transplant procedure may decrease the risk of relapse via deeper reducing of tumor burden before AHSCT [11]. The encouraging results were obtained for R/R FL patients who were treated with immuno-chemotherapy and in vivo purging before AHSCT with the 5-year PFS of 59 % [18].

Nevertheless, it was demonstrated that 3-year PFS after AHSCT for rituximab-naïve and rituximab-treated FL patients was comparable (72 vs. 75 %, respectively). The 3-year OS rates were 92 % for both arms. A trend for better 3-year PFS rate after AHSCT was found for rituximab-naïve patients receiving rituximab at progression versus those who did not receive rituximab at relapse (*p* = 0.07). AHSCT was beneficial in terms of PFS and OS for rituximab-treated FL patients if compared with those not receiving transplantation (*p* = 0.052) [16]. To date, there are no prospective studies that demonstrated the superiority of AHSCT over conventional chemotherapy in the rituximab era. Some single studies have shown a better disease control in AHSCT arm, but it did not translate into better OS [19]. It may be partially due to an increased risk of secondary cancers after autograft [16]; however, this presumption has not been shown by others [20], including us. It was concluded that radiotherapy as a preparative regimen before AHSCT might be responsible for the development of secondary malignancies and should be avoided [12]. In our study, radiotherapy was not a part of conditioning and no patient with secondary malignancy was detected after maximum of more than 18 years of follow-up. ^90^Y-radiolabeled ibritumomab tiuxetan was administered before AHSCT in five patients, and it was well tolerated. As the number of treated patients was low, it is difficult to draw far-reaching conclusions. Nevertheless, three out of the five treated patients are free of disease at the last contact.

In a majority of patients, the toxicity of transplant procedure was manageable and only one early posttransplant death due to severe pulmonary infection was observed in our study group. TRM at 100 days was 3 %. This finding is in line with the TRM rates provided by other groups [15, 21]. There was no difference in the PFS and OS rates regardless of FLIPI score and disease status at transplant. The similar results have been demonstrated by other groups [14]. Nevertheless, the following adverse risk factors for predicting a worse OS were identified: grade 3 FL, high FLIPI index at transplant and 3 or more chemotherapy lines before AHSCT [11]. This finding, however, requires confirmation in other studies.

## Conclusions

AHSCT for relapsed FL pretreated with rituximab remains a safe procedure with low TRM and long-term progression-free survival in about one-third of transplanted patients with no plateau. It seems reasonable to offer AHSCT for relapsed FL patients; however, its utility in the rituximab era requires to be elucidated.
